# Capillary Blood Recovery Variables in Young Swimmers: An Observational Case Study

**DOI:** 10.3390/ijerph19148580

**Published:** 2022-07-14

**Authors:** Robert Nowak, Konrad Rój, Andrzej Ciechanowicz, Klaudyna Lewandowska, Dorota Kostrzewa-Nowak

**Affiliations:** 1 Institute of Physical Culture Sciences, University of Szczecin, 17C Narutowicza Str., 70-240 Szczecin, Poland; dorota.kostrzewa-nowak@usz.edu.pl; 2 Student of ”Sports Diagnostics”, Faculty of Physical Education and Health, University of Szczecin, 40b Piastów Al., 70-240 Szczecin, Poland; kondziory@gmail.com; 3 Department of Clinical and Molecular Biochemistry, Pomeranian Medical University in Szczecin, 72 Powstańców Wlkp. Al., 70-111 Szczecin, Poland; andrzej.ciechanowicz@pum.edu.pl (A.C.); klaudyna.lewandowska@pum.edu.pl (K.L.)

**Keywords:** athletes, bilirubin, blood morphology, enzyme markers, ferritin

## Abstract

Sport diagnostics is still in pursuit of the optimal combination of biochemical and hematological markers to assess training loads and the effectiveness of recovery. The biochemical and hematological markers selected for a panel should be specific to the sport and training program. Therefore, the aim of this study was to evaluate the usefulness of selected biochemical and hematological variables in professional long-distance and sprint swimming. Twenty-seven participants aged 15–18 years took part in the study. Alanine aminotransferase (ALT), aspartate aminotransferase (AST), lactate dehydrogenase (LDH) and alkaline phosphatase (ALP) activities and creatinine (Cr), C-reactive protein (CRP), ferritin, total bilirubin (TB), direct bilirubin (DB) and iron concentrations were measured for 10 weeks and compared with the traditional sport diagnostic markers of creatine kinase (CK) activity and urea (U) concentration. Additionally, capillary blood morphology was analyzed. An effective panel should consist of measurements of CK and AST activities and urea, TB, DB and ferritin concentrations. These markers provide a good overview of athletes’ post-training effort changes, can help assess the effectiveness of their recovery regardless of sex or competitive distance and are affordable. Moreover, changes in ferritin concentration can indicate inflammation status and, when combined with iron concentration and blood morphology, can help to avoid iron deficiencies, anemia and adverse inflammatory states in swimmers.

## 1. Introduction

Training for competitive swimming has a long history of focusing on developing endurance and strength and improving specialized swimming techniques [1,2,3]. One of the key aspects of specialized training is determining the athlete’s threshold speed (i.e., speed at the anaerobic threshold). It is also important to increase the athletes’ phosphagen system capacity. Extending training process in the field of improving athletes’ aerobic abilities is important in building the predisposition to strength and speed efforts. It should be emphasized, however, that in swimming training it is important to shape both aerobic and anaerobic capacity, allowing to overcome water resistance. This allows the entire training macrocycle to be planned and the appropriate swimming speed to be selected in relation to intensity ranges [2,3,4,5]. In competitive swimming, it is necessary to use diagnostic tools and tests to determine the athlete’s efficiency and individualize training. These tools and tests can help maximize performance and monitor the impact of training loads on the athlete’s body by measuring markers such as lactic acid (LA) concentration immediately after or during training. Creatine kinase (CK) activity and urea (U) concentrations can also be used to monitor muscle damage and the energy cost of long lasting high-intensity training, respectively [6,7].

An optimal biochemical marker for assessing the degree of an athlete’s post-training recovery should respond only to exercise and react in a predictable and sustained manner. However, different sports require different training plans, trigger different responses and utilize energy pathways differently. Thus, there is a need for sport-specific blood test panels to determine the effectiveness of recovery. On the other hand, the high individual variability of CK activity as a marker makes it difficult for trainers to interpret the results. It should also be noted, that with an increase in the athlete’s conditioning, the maximum plasma CK activity in response to a training load may appear 24–36 h post-training [7,8,9,10,11]. Consequently, sport diagnostics require other biochemical markers. Some studies show that aminotransferases can be used as auxiliary diagnostic markers in sports, including swimming and replace the CK activity measurement commonly used by trainers, as they reflect the current level of muscle damage in a manner similar to plasma or serum CK activity [9]. However, the optimal solution seems to be to use them together, thus minimizing possible measurement errors and allowing the current state of the athletes’ liver to be diagnosed, remembering that initially alanine aminotransferase (ALT) and aspartate aminotransferase (AST) were used in laboratory diagnostics only in the case of suspected liver disease or damage [7,8,9,10,11]. Our previous study investigating long-term measurement of biochemical markers in elite soccer players indicated that AST, CK and lactate dehydrogenase (LDH) activity and creatinine (Cr) concentration, when analyzed together, could constitute a useful set of markers for monitoring the athletes’ recovery periods. AST, LDH and Cr seem to be particularly good markers because of the lower inter-individual variability of these parameters in comparison to CK [6,11,12].

Insufficient recovery time during training cycles can lead to fatigue and predispose young swimmers to mineral deficiency. Iron is one of several important micronutrients for athletes. Iron deficiency may be associated with the development of anemia and impaired sports performance [13,14]. For this reason measuring plasma iron and magnesium concentrations in swimmers could be an additional tool to monitor recovery effectiveness.

Athletes’ capability to improve their training performance can also be reflected in their blood morphology [15]. It is known that VO_2_ max correlates with O_2_ transport capacity, aerobic capacity and total hemoglobin concentration in athletes [16]. Our previous study also showed that changes in red blood cell distribution width (RDW), as well as other red blood cell-related parameters including mean corpuscular hemoglobin (MCH) and mean corpuscular hemoglobin concentration (MCHC), suggest that erythropoiesis may decrease at the end of training cycle [15]. White blood cell (WBC) count often decreases after a long-term training program [17,18]. On the other hand, our previous study found a post-exercise increase in WBC parameters related to the immune system’s response to the physical effort [19,20,21]. According to the literature, an important observation in the context of recovery profiles is that the baseline mean platelet volume (MPV) may be a predictor of endurance capacity [22,23]. Our previous study suggested that a training program focused on motor performance may cause a decrease in plateletcrit (PCT) associated with a decrease in platelet count (defined as a decrease in peak values) from the beginning to the end of the training program [15]. Increased fibrinolytic activity after physical effort is frequently observed in professional athletes [24]. Symptoms of platelet fatigue (i.e., functional impairment) have been observed in trained people during recovery [24,25]. In addition, it has been found that coagulation and fibrinolytic function in athletes are reduced or at least comparable to sedentary counterparts. It is possible that these changes are beneficial for physically active people, as they may be involved in protecting against thrombosis and adverse cardiovascular events [24,25,26]. Therefore, blood morphology assessment should be an important part of a panel used for monitoring recovery effectiveness in professional athletes. 

Competitive swimming is an individual sport discipline in which competitors are divided in training programs into three basic groups depending on their anatomical and physiological predisposition affecting the preferred start distance: sprint (50–100 m), middle-distance (200 m) and long-distance (400–1500 m) [27].

Monitoring changes in several peripheral blood markers is important for assessing athletes’ responses to training loads and the effectiveness of their recovery. This work demonstrates the use of routine laboratory diagnostic markers to evaluate the effectiveness of swimming athletes’ recovery compared to the traditional sport diagnostic markers of CK activity and U concentration. The main goal of this observational study was to compare the use of selected markers of clinical biochemistry (ALT, AST, LDH, ALP activities and Cr, CRP, ferritin, TB, DB) with the variability of CK activity and U concentration after recovery in champion-class sports swimmers.

## 2. Materials and Methods

### 2.1. Study Protocol and Participants

To evaluate the effectiveness of selected biochemical and hematological parameters as markers of recovery effectiveness in professional long-distance and sprint swimming, 27 participants aged 15–18 years and belonging to Polish swimming clubs were recruited for this observational study. The main inclusion criteria included required age, training experience, competitive distance and belonging to the championship-class. Participants not meeting inclusion criteria, not giving or retracting their consent to participate were excluded from the study. Taking the small number of participants into account, this work should be recognized as a case study. Participants were divided by sex and according to their competitive distance, giving male and female sprint (50–100 m) groups (MS and FS groups, respectively) and male and female long-distance (400–800 m) groups (ML and FL groups, respectively). All qualified female athletes were already after the pubertal spike. The general characteristics of the groups are presented in Table 1. Detailed participants’ training data regarding distance swam, water and dry-land trainings during the observation period are summarized in Supplementary Table S1. Changes in the biochemical and hematological parameters were observed for 10 weeks starting from the first day of the preparatory training stage after the summer holiday.

Eleven standard medical diagnostic markers including ALT, AST, LDH and alkaline phosphatase (ALP) activities and Cr, CRP, ferritin, total bilirubin (TB), direct (conjugated) bilirubin (DB) and iron concentrations were chosen to evaluate their usefulness as indicators of recovery effectiveness compared to the traditional sport diagnostic markers of CK activity and U concentration. Additionally, blood morphology indices were observed in all participants. 

All participants had been engaged in swimming training for at least 5 years and were classified as a junior or younger junior. The participants had no history of any metabolic syndrome according to the International Diabetes Federation description (diabetes, prediabetes, abdominal obesity, high cholesterol and high blood pressure) or cardiovascular disease (defined by the World Health Organization as disorders of the heart and blood vessels). Participants were non-smokers and refrained from taking any medications or supplements known to affect metabolism. Participants (and their parents if appropriate) were fully informed of any risks and discomfort associated with the experimental procedures before giving their written consent to participate. The study was approved by the Local Ethics Committee (approval number: 05/KB/VII/2019) in accordance with the Helsinki Declaration.

### 2.2. Blood Sampling

Fasted blood samples were obtained according to standard diagnostic procedures. Fingertip capillary blood collection systems (KABE-Labortechnik GmbH, Nümbrecht-Elsenroth, Germany) were used to collect blood samples for biochemical and hematological analyses using lithium heparin (2 capillaries per 200 μL of blood; cat. No. 077201) and ethylenediaminetetraacetic acid (EDTA; 1 capillary per 100 μL of blood; cat. No. 077103), respectively, as anticoagulants [28,29]. Capillary blood was collected between 6.00 am and 6.30 am after one day of recovery (i.e., a day where no training took place). 

### 2.3. Blood Analysis

All blood analyses were performed no longer than 60 min after collection. Blood plasma was used to determine CK, AST, ALT, ALP and LDH activities and U, Cr, TB, DB, CRP, ferritin and iron concentrations. All parameters were determined using a standardized diagnostic method, following the manufacturer’s instructions (BioMaxima S.A., Lublin, Poland or, in the case of ferritin, Quimica Clinica Aplicada S.A., Amposta, Spain). The biochemical tests were carried out using an Auto Chemistry Analyzer BM-100 (BioMaxima S.A., Lublin, Poland). All analyses were verified using a multiparametric control serum and normal (BioNorm) and high (BioPath) level control serums (BioMaxima S.A., Lublin, Poland). Blood morphology parameters were obtained using an automatic hematology analyzer ABX Micros 60 (Horiba ABX Sp. z o.o., Warsaw, Poland).

### 2.4. Statistical Analysis

All data are presented as the median (Q1–Q3) of the measurements taken. Statistical analyses were carried out using Statistica (version 13, TIBCO Software Inc., Palo Alto, CA, USA, 2017) or R (https://cran.r-project.org/ (accessed on 20 June 2022)), respectively. The normality of the data distribution within the subgroups was assessed using the Shapiro-Wilk test. Since the data was not normally distributed, nonparametric statistical analyses were carried out. Differences between the parameters obtained from females and males and between sprint and long-distance groups were analyzed using the Mann-Whitney U test. Differences between time points were analyzed using Friedman’s analysis of variance for repeated measures followed by Dunn’s post-hoc test with a Bonferroni correction. Correlations between variables were analyzed using Spearman’s rank correlation coefficient. For all analyses, a *p* value < 0.05 was considered significant.

General linear mixed model (GLMM) was used to examine whether laboratory parameters changed differently throughout the study in the short and long distance swimmers. The week of the study (time, treated as a categorical variable) and distance were fixed effects. Individuals were treated as random factors. The significance of the time and distance interaction was evaluated by comparing two models, with and without the interaction term using the likelihood ratio test. Results with FDR (False Discovery Rate)-adjusted *p* < 0.05 were considered statistically significant. General linear mixed models were fitted using lme4 package in R (https://cran.r-project.org/ (accessed on 20 June 2022)).

Statistical power of the tests was calculated using G* Power version 3.1.9.2 software (http://www.gpower.hhu.de (accessed on 15 October 2014)).

## 3. Results

### 3.1. Analysis of Selected Enzyme Activities

**Figure 1:**
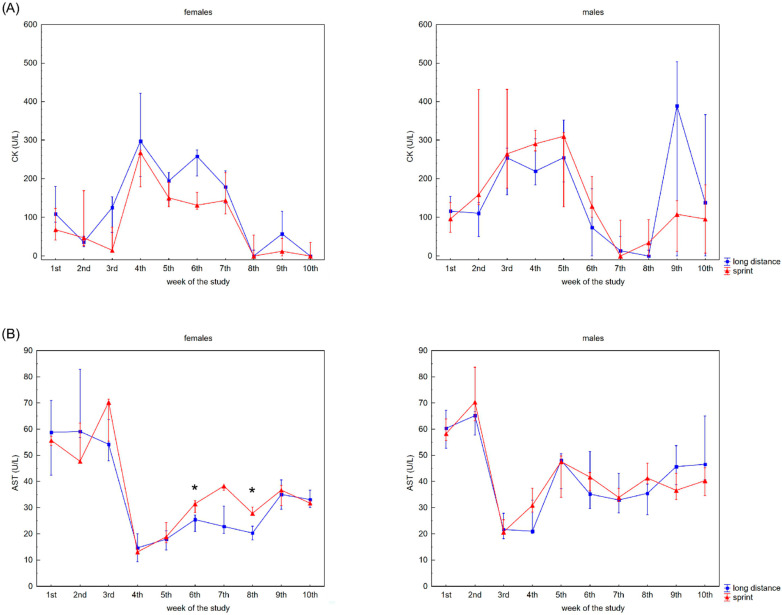

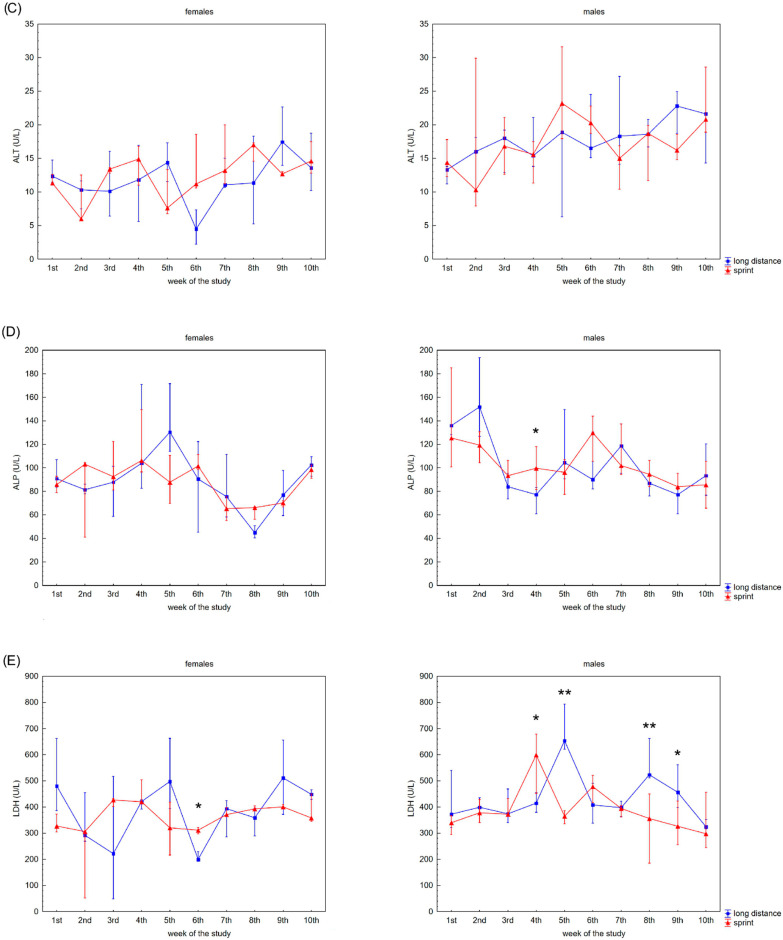
Capillary plasma median activity during recovery of: (**A**) creatine kinase (CK), (**B**) asparagine aminotransferase (AST), (**C**) alanine aminotransferase (ALT), (**D**) alkaline phosphatase (ALP), (**E**) lactate dehydrogenase (LDH) in female long-distance (FL) and sprint (FS) swimmers and male long-distance (ML) and sprint (MS) swimmers. The midpoint represents the median; whiskers represent the Q1–Q3 range. The significance of differences between the long distance and sprint groups were calculated using the Mann–Whitney U test. The statistical power of the test was equal to: 0.53 and 0.78 for AST in females in 6th and 8th week, respectively; 0.48 for ALP in males in 4th week; 0.93 for LDH in females in 6th week; 0.35, 0.99, 0.93 and 0.62 for LDH in males in 4th, 5th, 8th and 9th week, respectively. * *p*  <  0.05; ** *p*  <  0.01.

The recovery profile of CK, AST, ALT, ALP and LDH activities during the 10 weeks is presented in Figure 1. There were no significant differences between the female sprint and long-distance groups for CK, ALT and ALP activities (Figure 1A,C,D). In the male groups, no differences in CK, AST and ALT activities were observed (Figure 1A–C).

CK activity was negatively correlated with AST activity in the FS group, whilst there was a positive correlation between CK activity and AST and ALT activity in both male groups and LDH activity in the MS group (Table 2).

### 3.2. Analysis of Selected Metabolite Concentrations

**Figure 2:**
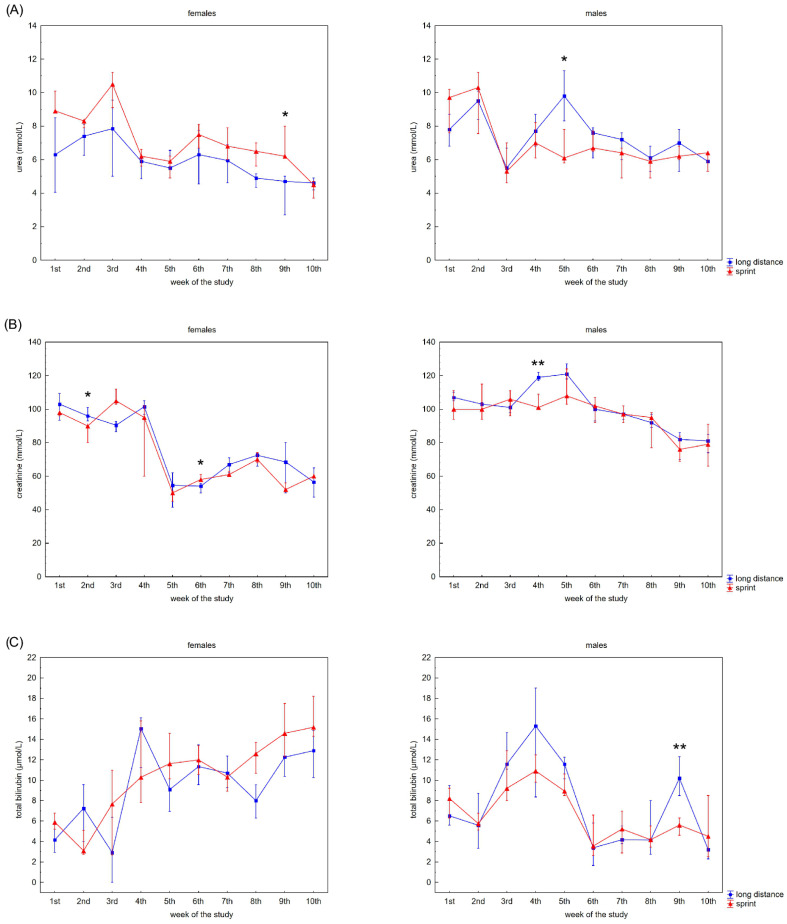

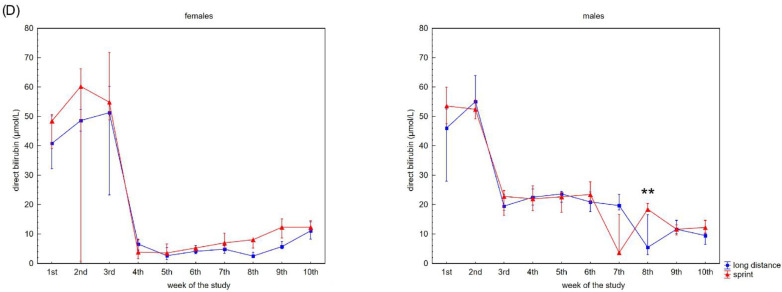
Capillary plasma median concentration during recovery of: (**A**) urea, (**B**) creatinine (Cr), (**C**) total (TB) and (**D**) direct (DB) bilirubin in female long-distance (FL) and sprint (FS) swimmers and male long-distance (ML) and sprint (MS) swimmers. The midpoint represents the median; whiskers represent the Q1–Q3 range. The significance of differences between the long distance and sprint groups was calculated using the Mann–Whitney U test. The statistical power of the test was equal to: 0.58 for urea in females in 9th week; 0.63 for urea in males in 5th week; 0.48 and 0.65 for Cr in females in 2nd and 6th week, respectively; 0.95 for Cr in males in 4th week; 0.94 for TB in males in 9th week; 0.98 for DB in males in 8th week. * *p*  <  0.05; ** *p* < 0.01.

Figure 2 presents the recovery concentrations of the metabolites in the swimmers’ capillary plasma. During the 10 weeks no significant differences were found between the sprint and long-distance groups, with the exception of TB and DB in males (Figure 2C,D). A decrease in recovery Cr and U concentrations was observed in all groups (Figure 2A,B), whilst the DB concentrations reduced after the 3rd and 2nd week in the female and male groups, respectively (Figure 2D). In contrast, TB concentrations in both female groups showed a tendency to increase during the study (Figure 2C). 

A negative correlation between CK activity and DB concentration was observed in the FS group (Table 2). U concentration correlated with AST activity and Cr, TB and DB concentrations in female swimmers (Table 2). In the male groups, correlations between CK activity and U, Cr, TB and DB concentrations and between U concentration and AST, LDH activity and DB concentration were found (Table 2). 

### 3.3. Analysis of Selected Pro-Inflammatory Proteins and Ions

**Figure 3:**
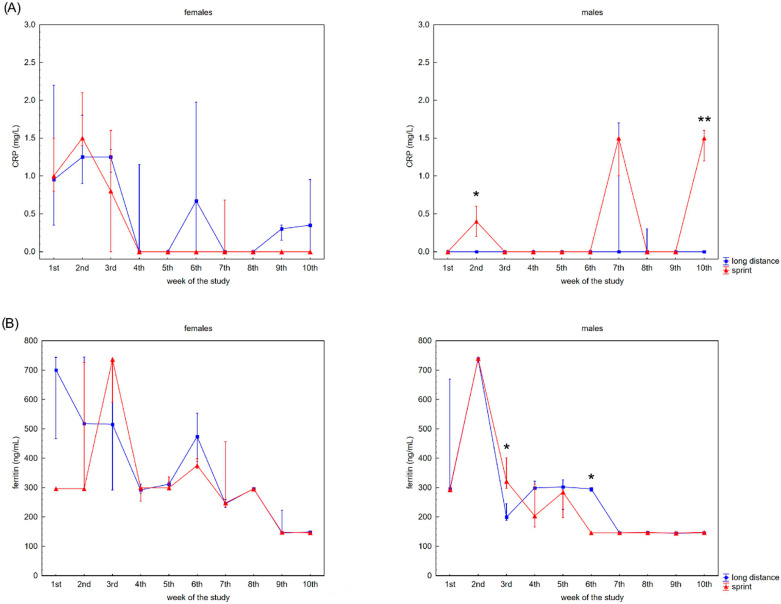

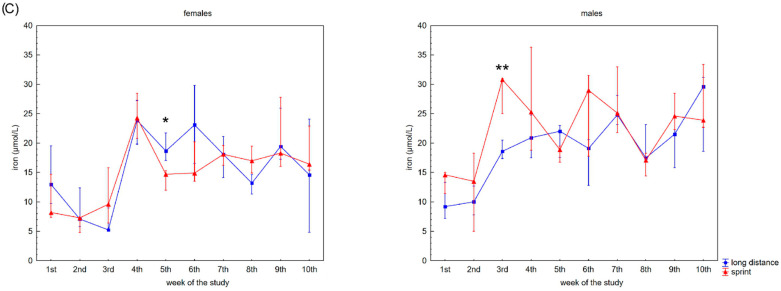
Capillary plasma median concentration during recovery of: (**A**) C-reactive protein (CRP), (**B**) ferritin, and (**C**) iron in female long-distance (FL) and sprint (FS) swimmers and male long-distance (ML) and sprint (MS) swimmers. The midpoint represents the median; whiskers represent the Q1–Q3 range. The significance of differences between the long distance and sprint groups was calculated using the Mann–Whitney U test. The statistical power of the test was equal to: 0.68 and 0.99 for CRP in males in 2nd and 10th week, respectively; 0.84 and 0.65 for ferritin in males in 3rd and 6th week, respectively; 0.31 for iron in females in 5th week; 0.87 for iron in males in 3rd week. * *p*  <  0.05; ** *p*  <  0.01.

The swimmers’ recovery profiles for CRP concentration are presented in Figure 3A,B. Although there were slight alterations in this variable in the female groups over the 10 weeks, post-hoc analyses did not confirm any significant differences. A probable explanation for this is the correlation between U and CRP concentrations observed in the FS and FL groups (Table 2). 

Ferritin differed significantly in the male groups during the observation time (Figure 3B). The recovery concentration of ferritin positively correlated with CK and AST activity in the female and male sprint groups, respectively. There was also a positive correlation between ferritin and U in all groups. A negative correlation between ferritin and iron concentrations was found in the male groups, with negative correlations between ferritin concentration and ALT activity and ferritin concentration and TB concentration found in the FL group (Table 2). 

Iron recovery plasma concentrations showed a tendency to increase regardless of sex and competitive distance during the 10 weeks (Figure 3C). Iron concentration correlated positively with CK activity in the female groups. There was also a negative correlation between iron concentration and U concentration in the FS group (Table 2).

### 3.4. Analysis of Capillary Blood Morphology

The changes in white blood cell (WBC) counts were significant in all groups, excluding the FL group (Table 3 and Table 4). The highest median WBC count in the FS group was found in the 4th week, whilst at the end of the observation period the median WBC count was similar to that at baseline during the 1st week (Table 4). Also, in the ML group the highest median WBC count was found in the 4th week (Table 4). Significant differences for this variable between the male sprint and long-distance groups were only observed in the 3rd week (Table 4). Regarding the WBC subsets of lymphocytes (LYM), monocytes (MON) and granulocytes (GRA), the pattern of significant changes is different in each group for both the percentage and absolute counts. The highest median LYM percentage in female swimmers was in the 2nd week in the FS group and in the 1st week in the FL group, whilst LYM absolute count was highest in the 4th week in the FS group and highest in the 5th week in the FL group (Table 3). The highest median percentage of LYM was observed in the 2nd week in the ML group and in the 8th week in the MS group. Taking LYM absolute count into account, the highest values were noted in the 4th week in both male groups (Table 4). When comparing LYM values in females and males, significant differences were found in the 2nd week in long-distance swimmers (% LYM, *p* = 0.025), whilst in the sprint groups, differences in % LYM were observed in the 9th week (*p* = 0.045) and in the 4th week (*p* = 0.033) for LYM absolute count. No differences in MON (both percentage and absolute count) between distance groups in females and males were observed, but there were significant differences in % MON in the 7th (*p* = 0.025) and 10th (*p* = 0.007) weeks in the long-distance groups and in the 3rd (*p* = 0.014), 5th (*p* = 0.045), 6th (*p* = 0.011), 8th (*p* = 0.020), 9th (*p* = 0.008) and 10th (*p* = 0.003) weeks in the sprint groups. When analyzing sex differences in MON absolute counts, there were significant changes in the 3rd (*p* = 0.017), 7th (*p* = 0.011) and 10th (*p* = 0.007) weeks of observation in the long-distance groups and in the 9th (*p* = 0.004) and 10th (*p* = 0.003) weeks in the sprint groups. The fluctuation patterns of GRA percentage were different from absolute count values in all groups (Table 3 and Table 4). The highest median values of % GRA were found in the final week in female groups (Table 3) and in the 9th and 7th weeks of in the MS and ML groups, respectively (Table 4). 

The highest red blood cell (RBC) counts in the female groups were observed in the middle of the study (4th–6th week; Table 3). In male swimmers, RBC count fluctuated, with the highest values at the beginning and end of the study. A similar pattern was observed for hemoglobin (Hb) concentration. Different patterns of changes in the RBC-related parameters of hematocrit (HCT), mean corpuscular volume (MCV), mean corpuscular hemoglobin (MCH), mean corpuscular hemoglobin concentration (MCHC) and RDW were found in all groups (Table 3 and Table 4).

There was no significant difference in platelet absolute count (PLT) between sprint and long-distance female swimmers (Table 3). The lowest median PLT value was observed in the 10th week in the ML group, whilst in the MS group, the median PLT value at this time point was the highest (Table 4). Different patterns of alterations in the platelet-related variables of mean platelet volume (MPV), PCT and platelet distribution width (PDW) were observed during the study (Table 3 and Table 4).

### 3.5. Mathematical Modeling

The results of general linear mixed model (GLMM) used to examine whether laboratory parameters changed differently throughout the study in the short and long distance swimmers are presented in Supplementary Table S2. Interestingly, FDR-adjusted *p* was significant only for MCHC in the case of females. In the case of male athletes more variables revealed to be statistically significant in this mathematical model. 

## 4. Discussion

The optimal balance between training and recovery must be maintained to maximize physical and specific performance and is why tools to monitor recovery should be used in professional swimming. There are numerous studies describing new methods for planning training programs and testing athletes’ performance [1,4,9]. Monitoring biochemical and hematological variables would help to create a more individualized and specific recovery profile for an athlete. It must be pointed out that intergroup comparisons performed in regards of distance covered showed that in most cases there were no significant changes between distance groups. Moreover, the tendencies of changes in analyzed variables were very similar regardless the distance covered by swimmers.

### 4.1. Enzymatic Markers in Capillary Blood Recovery Profiles of Swimmers

CK activity constitutes the most common enzymatic marker used in sport diagnostics, because physical effort can trigger rhabdomyolysis and the release of muscle cell content [6,9,30]. The common problem with this parameter is its huge variation in baseline values, with reference ranges for the general population dependent on age, sex, race, muscle mass, physical activity and climatic conditions [15,31]. Metabolic homeostasis is achieved approximately 48–72 h after physical effort [32] and for this reason CK activity is the most commonly used recovery marker. Although the literature provides CK activity reference ranges for athletes (males: 82–1083 U/L; females: 47–513 U/L) [9,33], higher values have been reported [9]. The values observed in our study are within these reference ranges, but they do exceed general population reference ranges. Different sport disciplines are characterized by different exercise loads, and this—together with the inter- and intra-personal variability reported—makes it advisable to extend routine sport diagnostics to include other clinical parameters. It should also be noted that the observed fluctuations in CK activity result largely from the fact that the research was conducted at the beginning of the training period, which contributes to the high variability of the value of this sports diagnostic maker. Interchangeability between individuals and individually increasing training loads contribute to the observed fluctuations in results.

In the present study, the changes in AST, ALT, ALP and LDH activities observed in the capillary blood of swimmers during recovery varied depending on the athlete’s competitive distance and sex. It is commonly accepted that the increase in AST and ALT activity in professional athletes is associated with the release of these enzymes from muscle cells rather than liver pathology [6,8,11,34]. The results of our study showed that LDH activity slightly correlated with CK activity in the MS group only, whilst ALT and AST activity correlated with CK activity in both male groups and the FS group. Taking the economic and practical aspects of clubs’ daily sport diagnostics into account, our study indicated that, in the case of swimmers, measuring athletes’ CK activity should be combined with AST activity regardless of sex and competitive distance. AST activity not only correlated with CK activity, but also may be important for making decisions about training and recovery due to the high variability in resting CK values in athletes. Also, AST activity correlated with athletes’ U concentration regardless of their sex. Therefore, AST can be used as a decisive marker if there are doubts whether the increased concentration of U is the result of changes in the athlete’s diet or is a consequence of a completed training loads.

### 4.2. Selected Metabolic Substrates in Capillary Blood Recovery Profiles of Swimmers

Another commonly assessed sport diagnostic marker is U. In contrast to CK activity, its blood concentration is more stable and values for both athletes and the general population are within the same range. It is postulated that a low concentration of U indicates the ability to increase the training load in football players and that it should be used to assess training loads [6,35]. However, U concentration exceeding the upper limit of the reference range might be a predictor of more efficient energy metabolism, where protein breakdown for gluconeogenesis is higher [6,12,36,37], being in this case an undesirable phenomenon. 

The study of male football players found a statistically significant correlation between post-exercise plasma Cr levels and training experience and between plasma Cr plasma levels during recovery and weekly training frequency. Similar to the present study, a correlation between plasma Cr levels and CK activity in samples taken during recovery was found [38,39]. The results of our study seem to indicate a similar relationship between Cr levels and CK activity in male swimmers. 

U also appears to provide a good indication of fatigue levels in male swimmers. It is well known that athletes’ blood Cr concentration is a fairly stable variable dependent on their sex [38,40,41]. Fluctuations of Cr concentration are commonly related to training load, aerobic/anaerobic metabolism, competition length and the stage of a competitive season (possibly due to changes in an athlete’s muscle mass during the season) [8,9,40]. Moreover, in the present study, U concentration correlated with CK activity. The results of our study indicate that these two markers are convergent in determining the microdamages of skeletal muscles and the energy costs resulting from training loads.

Measuring different blood forms of bilirubin, including unconjugated, free and albumin bound conjugated, DB and TB may provide critical information for the diagnosis of many diseases and metabolic disorders [42]. It is also valuable in sport diagnostics [8]. Generally, increases in the concentration of bilirubin in athletes’ serum or plasma is related with hemolysis [8,43] (e.g., footstrike hemolysis, mechanical damage to RBCs during continuous muscle contractions, continuous exposure to high-oxygen flux causing oxidative damage and perturbation of osmotic homeostasis which might render RBCs more susceptible to membrane damage during their transit through the microcirculation [43,44,45]). Moreover, literature has suggested that bilirubin is a potential anti-inflammatory and antioxidant [46,47]. In the present study, it was observed that both TB and DB increased at the beginning of the training program and after 2–3 weeks these values were significantly lower than baseline concentrations regardless of the swimmers’ sex and competitive distance. This suggests that the capillary concentration of both TB and DB during recovery can be a marker of training adaptation following a break. TB and DB concentrations during recovery can be used to assess the extent of training that the swimmer can engage in without causing excessive post-training intravascular hemolysis. 

### 4.3. Selected Pro-Inflammatory Proteins and Ions in Capillary Blood Recovery Profiles of Swimmers

It is well known that post-training micro-damage of muscle tissue is related to local inflammatory processes. Is must be pointed out that an inflammatory state can have a positive immunomodulatory effect. Yet, it can be excessive and lead to negative effects [48,49,50,51,52,53,54,55,56]. Balancing the positive and negative effects of an inflammatory state is important for improving training program effectiveness. Consequently, it is important to monitor the concentration of pro-inflammatory proteins in athletes’ blood. The list of pro-inflammatory markers is long and includes interleukin-6 (IL-6), interleukin-1 beta (IL-1β), CRP and tumor necrosis factor alpha (TNFα) [19,57,58,59]. However, the finances of sport clubs’ may require sport diagnosticians to choose one or two proteins to monitor inflammatory processes during the training program. In such circumstances, CRP is the protein of choice because it plays an essential role in acute and chronic stress triggered by training or illness-related changes in the organism [58,60]. Our study found evidence that CRP is not a universal pro-inflammatory marker in swimmers. According to the literature, CRP concentration can be related to athletes’ physical fitness [61,62]. However, it must be pointed out that acute exercise may cause an increase [59,63,64] or no change [65] in CK activity. Even though CRP concentrations were in line with CK activity in football players [58], a similar observation was noticed only in male long-distance swimmers. Our study showed that CRP concentration correlated with U concentration, but only in the female groups. It could explain the lack of significant differences in CRP profile in the female group. It seems that in the present study, regardless of swimmers’ sex and competitive distance, CRP was not related to the systemic response to exercise-induced inflammation that stimulates muscular regeneration. This is why we postulate that CRP should not be the marker of first choice to be used when assessing recovery processes in swimmers. 

Ferritin is a protein related to iron metabolism, but the most important role of this protein in sport diagnostics is related to its pro-inflammatory role [66,67,68,69,70]. Serum ferritin is widely recognized as an acute phase reactant and marker of acute and chronic inflammation. It is nonspecifically elevated in a wide range of inflammatory conditions [71,72], but is not often associated with physical effort and inflammation. It is suggested that an increase in ferritin concentration may be linked with relative iron deficiency and inflammatory illness, and malignancy is developed as a defense mechanism to restrict serum iron from utilization by pathogens and tumors [71,73,74]. The ferritin concentration during recovery observed in our study decreased as a function of time in both sexes regardless of competitive distance, whilst iron concentrations increased during the same period. These observations support the hypothesis that during inflammatory states there is increased total iron storage with increased ferritin an indicator of inflammation. Although elevated, the iron is not available for hematopoiesis due to its sequestration (a phenomenon known as anemia of inflammation) [71,73]. The changes in iron and ferritin concentrations described above are reflected in RBC parameters measured as part of the blood morphology recovery profile.

### 4.4. Morphology of Capillary Blood Recovery Profiles of Swimmers

It is well known that different types and durations of physical effort affect hematologic parameters differently. Absolute RBC counts and RBC-related parameters (e.g., MCH, MCHC) provide important information about the general health of athletes [9,75,76]. The fluctuations observed during the present study were within the range of general population reference limits. Post-training effort changes observed in RDW and other RBC-related parameters (e.g., MCH, MCHC) may suggest increased erythropoiesis during the training process [15]. However, this was not observed in the samples collected from the swimmers in the present study. 

One of the most common post-training effort observations regarding athletes’ blood is exertional leukocytopenia, which usually occurs 30–60 min after completing training [21,77,78]. WBC absolute count is often reduced after a long-term training program [79,80]. Post-training effort changes in WBCs and their subsets are often related to the migration of WBCs between the vascular bed and the extracellular space following increased blood flow and increases in epinephrine and cortisol concentrations [81]. It is commonly accepted that WBCs and their subsets reflect an athlete’s general health. Thus, the results of our study suggest that the training loads undertaken by the swimmers triggered positive adaptations. 

Our previous study indicated that a motor performance training program caused a decrease in PCT [15]. Such decreased coagulation and fibrinolytic function in recovery might be a beneficial for athletes due to the protection it provides against thrombosis and adverse cardiovascular events that might occur during exercise [23,24]. Therefore, monitoring of platelet parameters is an easy way to control an organism’s fibrinolytic activity after exercise. In this study, no signs of platelet fatigue (i.e., impaired functionality) were observed in the swimmers. 

### 4.5. Mathematical Modeling

The results of general linear mixed model (GLMM) is not in line with raw data analysis. The reason for this phenomenon is small sample size. However, analyzing GLMM results for female and male athletes, it can be noticed that even a slight increase in the sample size improves the quality of the model.

### 4.6. Limitations of the Study

The present study has several limitations, including the small number of participants in each group. Recruiting more participants would strengthen our observations and conclusions and enable achieve more accurate mathematical model for such data. However, a limited number of swimmers matched the required age, training experience and competitive distance. Taking blood samples from the fingertip instead of venous blood sampling may be questionable. We chose such fingertip blood sampling as it was less invasive and easier to perform, and taking venous blood samples for 10 consecutive weeks is not recommended.

## 5. Conclusions

There is no doubt that by analyzing a greater number of blood parameters, more information for trainers can be obtained. However, sport clubs’ finances may mean that sport diagnosticians need to choose only the most important and informative parameters for monitoring. Our study showed that an optimal panel should combine measurements of CK and AST activities and U, TB, DB and ferritin concentrations. These parameters, regardless of sex and competitive distance, provide information about athletes’ training effort and help to assess the effectiveness of their recovery. It must be emphasized that both TB and DB concentrations during recovery can be used to assess swimmers’ adaptations to training and to program appropriate training loads without inducing excessive post-training intravascular hemolysis. Changes in ferritin concentration can reflect basic inflammation status, and when combined with iron concentration and blood morphology, can help to avoid iron deficiencies, anemia and adverse inflammation states in swimmers. Moreover, ferritin concentration seems to be an even more informative marker than CRP.

## Figures and Tables

**Table 1:** Participant characteristics.

Variable	FS Group	FL Group	MS Group	ML Group
	(N = 5)	(N = 4)	(N = 9)	(N = 9)
Age (years)	17 (15–18)	16 (15–18)	16 (15–18)	17 (16–18)
Height (cm)	180 (175–185)	175 (168–178)	183 (175–187)	188 (176–189)
Weight (kg)	64.0 (60.0–64.5)	61.0 (56.5–68.2)	67.6 (65.0–70.0)	78.0 (76.0–82.0)
Body Mass Index (kg/m^2^)	21.1 (18.3–22.0)	20.3 (19.8–21.7)	20.8 (20.1–21.5)	22.5 (22.1–24.8)
Average FINA ^1^ points	708 (658–794)	664 (656–691)	664 (610–858)	665 (600–817)
Length of training experience (years)	9.0 (9.0–11.0)	10.5 (8.5–12.0)	9.0 (8.0–9.0)	10.0 (7.0–11.0)
Weekly training volumes (h)	18.0 (16.5–20.0)	23.5 (19.0–28.5)	18.0 (18.0–20.0)	22.5 (20.0–27.0)
Weekly distance swam (km)	35.0 (14.5–53.0)	34.5 (20.0–51.4)	33.7 (19.0–51.0)	51.2 (22.0–61.2)
Weekly water training sessions	9 (4–11)	10 (5–12)	8 (4–11)	10 (5–12)
Weekly dry-land trainings (h)	5 (2–9)	7 (4–10)	8 (4–12)	6 (2–12)

^1^ FINA—International Swimming Federation (Fédération Internationale de Natation). The table presents median (Q1–Q3) values, except for age, average FINA points, weekly distance swam, weekly training sessions and weekly dry-land training, where median (min–max) is presented. N—number of participants.

**Table 2:** Coefficients of correlation between the analyzed variables of each group.

Variable	FS Group		FL Group		MS Group		ML Group	
	(N = 5)		(N = 4)		(N = 9)		(N = 9)	
	R	*p*	R	*p*	R	*p*	R	*p*
**CK (U/L) &**								
AST (U/L)	−0.31	0.028	−0.28	0.078	0.32	0.002	0.29	0.006
ALT (U/L)	−0.22	0.131	−0.06	0.729	0.41	<0.0001	0.39	0.001
ALP (U/L)	0.05	0.747	0.19	0.236	0.02	0.865	−0.29	0.006
LDH (U/L)	−0.03	0.851	−0.12	0.446	0.25	0.017	0.01	0.925
U (mmol/L)	−0.06	0.691	0.17	0.303	0.26	0.013	0.32	0.002
Cr (mmol/L)	−0.15	0.303	0.02	0.885	0.31	0.003	0.30	0.004
TB (µmol/L)	−0.01	0.976	0.22	0.174	0.25	0.015	0.34	0.001
DB (µmol/L)	−0.31	0.030	−0.17	0.289	0.22	0.041	0.09	0.410
CRP (mg/L)	−0.12	0.395	−0.09	0.587	−0.13	0.237	−0.35	0.001
ferritin (ng/mL)	0.33	0.019	0.11	0.489	0.30	0.005	0.07	0.482
Fe (µmol/L)	0.29	0.039	0.40	0.010	0.12	0.273	0.16	0.122
**U (mmol/L) &**								
AST (U/L)	0.63	<0.0001	0.29	0.071	0.54	<0.0001	0.35	0.001
ALT (U/L)	−0.24	0.092	−0.18	0.265	0.09	0.397	0.21	0.049
ALP (U/L)	−0.04	0.800	−0.03	0.833	0.15	0.168	0.18	0.092
LDH (U/L)	−0.06	0.686	−0.03	0.852	0.06	0.601	0.21	0.047
Cr (mmol/L)	0.49	0.001	0.15	0.369	0.07	0.501	0.25	0.016
TB (µmol/L)	−0.43	0.002	−0.15	0.346	−0.08	0.460	0.14	0.190
DB (µmol/L)	0.38	0.007	0.43	0.006	0.46	<0.0001	0.37	0.001
CRP (mg/L)	0.45	0.001	0.31	0.047	0.11	0.290	−0.03	0.746
ferritin (ng/mL)	0.38	0.006	0.41	0.008	0.34	0.001	0.24	0.018
Fe (µmol/L)	−0.36	0.009	0.01	0.990	−0.14	0.180	−0.01	0.912
**ferritin (ng/mL) &**								
AST (U/L)	0.34	0.017	0.11	0.491	0.33	0.001	0.21	0.052
ALT (U/L)	−0.17	0.247	−0.50	0.001	−0.08	0.460	−0.18	0.086
ALP (U/L)	0.17	0.232	0.03	0.865	0.13	0.211	0.16	0.126
LDH (U/L)	0.06	0.662	−0.14	0.398	0.05	0.633	0.11	0.303
Cr (mmol/L)	0.32	0.022	0.25	0.112	0.37	0.001	0.49	<0.0001
TB (µmol/L)	−0.12	0.392	−0.43	0.005	0.19	0.071	0.18	0.093
DB (µmol/L)	0.20	0.163	0.20	0.218	0.50	<0.0001	0.63	<0.0001
CRP (mg/L)	0.25	0.078	0.24	0.129	−0.12	0.262	−0.14	0.183
Fe (µmol/L)	−0.25	0.076	−0.09	0.567	−0.23	0.025	−0.37	0.001

The analysis of correlations between analyzed variables was performed using Spearman’s rank correlation coefficient. CK—creatine kinase; AST—aspartate aminotransferase; ALT—alanine aminotransferase; ALP—alkaline phosphatase; LDH—lactate dehydrogenase; U—urea; Cr—creatinine; TB—total bilirubin; DB—direct bilirubin; CRP—C-reactive protein; Fe—iron.

**Table 3:** Female participants’ capillary blood recovery morphology in sprint (FS) and long-distance (FL) groups during 10 weeks of training.

**Variable**	**1st Week**	**2nd Week**	**3rd Week**	**4th Week**	**5th Week**	**6th Week**	**7th Week**	**8th Week**	**9th Week**	**10th Week**
**FS Group**										
WBC (10^9^/L)	4.1	5.4	5.5	8.9	5.1	5.2	4.3	5.6	4.8	6.9
	(3.9–4.7)	(5.4–5.7)	(5.0–7.3)	(7.9–9.9)	(5.0–5.8)	(4.7–6.6)	(4.3–5.1)	(5.4–5.8)	(4.7–5.2)	(5.7–8.8)
RBC (10^12^/L)	4.3	4.5	4.7	5.2	5.0	5.2	4.9	4.4	4.4	4.4 *
	(4.3–4.4)	(4.4–4.5)	(4.5–4.8)	(4.9–5.5)	(4.8–5.1)	(5.2–5.3)	(4.8–5.0)	(4.3–4.5)	(4.3–4.6)	(4.3–4.5)
Hb (mmol/L)	7.4	7.7	7.3	9.8	8.7	9.5	9.1	7.5	7.9	7.5 *
	(7.2–8.0)	(7.7–7.9)	(8.0–8.1)	(9.7–10.3)	(8.6–9.0)	(9.2–9.8)	(8.9–9.2)	(7.2–7.5)	(7.6–7.9)	(7.3–7.6)
HCT (%)	39.8	41.0	43.6	47.0	42.7	46.7	44.5	41.9	41.5	40.5
	(39.6–41.5)	(41.0–41.2)	(41.8–43.6)	(46.6–51.0)	(42.5–45.1)	(43.9–46.8)	(44.3–45.7)	(40.6–42.7)	(39.8–41.6)	(39.3–40.7)
PLT (10^9^/L)	201	287	284	302	275	253	245	226	237	230
	(191–230)	(277–287)	(275–289)	(297–309)	(206–287)	(238–262)	(245–248)	(208–345)	(230–275)	(220–269)
PCT (10^−2^ L/L)	0.15	0.22	0.21	0.20	0.21	0.18	0.17	0.17	0.19	0.19
	(0.15–0.16)	(0.18–0.22)	(0.20–0.22)	(0.20–0.21)	(0.15–0.22)	(0.17–0.18)	(0.16–0.17)	(0.16–0.24)	(0.18–0.21)	(0.18–0.21)
MCV (fL)	92	92	91	92	89	89	89	95	93	90
	(92–94)	(91–93)	(90–93)	(90–94)	(86–89)	(89–91)	(88–90)	(95–95)	(90–93)	(89–92)
MCH (fmol)	1.72	1.75	1.70	1.86 *	1.81	1.87	1.81	1.70	1.71	1.68
	(1.72–1.80)	(1.71–1.77)	(1.69–1.81)	(1.86–1.93)	(1.69–1.83)	(1.81–1.88)	(1.80–1.82)	(1.68–1.71)	(1.71–1.79)	(1.66–1.73)
MCHC (mmol/L)	18.7	18.9 *	18.8 *	20.7	20.3	20.6	20.2	17.7	19.1	18.5 *
	(18.7–18.7)	(18.8–19.2)	(18.7–19.4)	(20.5–20.7)	(19.9–20.5)	(20.4–20.8)	(20.2–20.5)	(17.6–17.9)	(19.0–19.2)	(18.5–18.8)
RDW (%)	12.1	11.4	12.0	12.9	13.6	13.3	13.6	11.8	12.4	12.0 *
	(11.7–12.3)	(11.4–11.5)	(11.9–12.4)	(12.8–13.4)	(13.3–13.9)	(12.9–13.4)	(13.2–13.6)	(11.4–12.0)	(12.0–12.5)	(11.7–12.3)
MPV (fL)	7.8	7.8	7.3	7.2 *	7.5	7.2	6.7	7.5	8.1	8.0
	(7.5–7.9)	(7.3–7.8)	(7.1–7.3)	(6.6–7.3)	(7.5–7.9)	(7.2–7.6)	(6.6–6.8)	(7.1–7.6)	(8.0–8.1)	(7.8–8.1)
PDW (%)	15.5	15.4	15.0	16.0	15.4	15.6	16.7	15.9	15.1	15.4
	(15.5–15.7)	(15.4–15.5)	(14.2–15.9)	(15.8–16.7)	(15.0–15.7)	(15.5–15.7)	(15.3–17.1)	(15.3–16.1)	(14.1–15.1)	(15.0–16.1)
LYM (%)	39.9	47.7 *	43.3 *	39.7	40.9	38.6	45.3	44.6	34.5	24.5 *
	(39.7–42.6)	(46.0–50.7)	(32.4–43.9)	(38.7–41.5)	(37.7–45.1)	(36.6–39.2)	(37.0–46.8)	(40.2–46.4)	(32.7–35.3)	(19.7–29.4)
MON (%)	7.9	7.7	7.5	4.4	4.3	4.7	3.8	11.9	6.3	5.6
	(7.8–8.4)	(7.7–8.7)	(7.0–8.0)	(4.3–6.4)	(4.1–5.0)	(4.3–5.3)	(3.8–7.3)	(7.0–12.2)	(5.9–7.0)	(4.1–7.0)
GRA (%)	51.8	45.9 *	49.7	54.0	55.0	56.7	45.9	47.6	59.1	71.3 *
	(49.6–51.9)	(40.6–46.3)	(46.7–60.1)	(53.9–56.9)	(49.9–58.0)	(56.5–58.1)	(41.6–51.1)	(46.6–48.6)	(58.8–59.2)	(65.1–74.6)
LYM (10^9^/L)	1.8	2.5	2.3	3.5	2.0	2.1	2.0	2.1	1.8	1.6
	(1.5–1.9)	(2.4–3.0)	(1.9–2.5)	(3.2–3.8)	(1.8–3.1)	(1.7–2.5)	(1.9–2.3)	(2.0–2.5)	(1.5–1.9)	(1.5–1.8)
MON (10^9^/L)	0.3	0.4	0.3	0.4	0.2	0.3	0.2	0.6	0.3	0.3
	(0.3–0.3)	(0.4–0.5)	(0.3–0.5)	(0.3–0.4)	(0.2–0.2)	(0.2–0.3)	(0.1–0.3)	(0.3–0.7)	(0.3–0.4)	(0.2–0.4)
GRA (10^9^/L)	2.2	2.6	3.5	5.7	2.9	2.8	2.3	2.7	3.0	5.0
	(2.0–2.5)	(2.6–2.6)	(2.9–3.6)	(4.4–5.8)	(2.9–3.0)	(2.8–3.8)	(2.2–2.4)	(2.6–2.8)	(2.6–3.1)	(3.9–6.7)
**Variable**	**1st Week**	**2nd Week**	**3rd Week**	**4th Week**	**5th Week**	**6th Week**	**7th Week**	**8th Week**	**9th Week**	**10th Week**
**FL Group**										
WBC (10^9^/L)	5.5	7.4	6.7	7.2	9.6	5.9	5.4	5.3	5.4	6.9
	(4.9–7.4)	(5.0–8.4)	(6.0–8.6)	(6.2–8.6)	(8.0–10.3)	(5.4–6.2)	(4.6–5.7)	(4.2–6.6)	(4.8–6.4)	(5.7–8.8)
RBC (10^12^/L)	4.2	4.3	4.5	5.0	5.4	4.9	4.8	4.3	4.3	4.4
	(4.1–4.3)	(4.1–4.8)	(4.2–4.7)	(4.8–5.3)	(5.2–5.5)	(4.8–5.1)	(4.7–5.0)	(4.2–4.5)	(4.2–4.3)	(4.3–4.5)
Hb (mmol/L)	7.5	7.3	8.1	8.8	9.6	9.4	8.7	7.2	7.2	7.5
	(7.3–7.8)	(7.1–7.9)	(7.5–8.5)	(8.5–9.5)	(8.9–9.8)	(9.1–9.8)	(8.4–9.1)	(6.9–7.6)	(6.9–7.5)	(7.3–7.6)
HCT (%)	38.9	39.7	42.0	43.7	47.5	44.8	43.3	40.2	39.6	40.5
	(37.8–39.1)	(38.5–42.8)	(39.1–44.0)	(42.1–46.7)	(44.6–48.6)	(43.8–46.7)	(42.5–44.7)	(38.5–41.5)	(38.3–40.6)	(39.3–40.7)
PLT (10^9^/L)	174	230	308	291	326	242	227	267	233	230
	(140–226)	(200–254)	(253–329)	(251–320)	(313–347)	(229–264)	(185–270)	(175–336)	(215–265)	(220–269)
PCT (10^−2^ L/L)	0.14	0.18	0.22	0.22	0.24	0.16	0.15	0.19	0.19	0.19
	(0.11–0.17)	(0.15–0.19)	(0.20–0.23)	(0.19–0.25)	(0.22–0.25)	(0.15–0.18)	(0.13–0.18)	(0.13–0.23)	(0.17–0.23)	(0.18–0.21)
MCV (fL)	91	90	92	88	89	91	90	92	92	90
	(90.5–92.0)	(89–93)	(89–96)	(85–90)	(83–92)	(89–94)	(87–92)	(91–94)	(90–93)	(89–92)
MCH (fmol)	1.79	1.68	1.77	1.77	1.80	1.98	1.81	1.68	1.68	1.68
	(1.72–1.85)	(1.64–1.74)	(1.74–1.85)	(1.73–1.83)	(1.63–1.88)	(1.9–10.2)	(1.73–1.87)	(1.66–1.69)	(1.63–1.72)	(1.66–1.73)
MCHC (mmol/L)	19.5	18.5	19.4	20.3	20.1	20.9	20.0	18.1	18.2	18.5
	(18.7–20.5)	(18.4–18.6)	(19.2–19.4)	(20.2–20.3)	(19.6–20.5)	(20.7–21.0)	(19.7–20.4)	(17.7–18.6)	(18.0–18.4)	(18.5–18.5)
RDW (%)	12.9	12.1	11.6	13.5	13.5	13.0	14.0	12.1	12.3	12.0
	(11.8–13.5)	(11.4–13.0)	(11.2–12.0)	(13.4–14.5)	(13.0–15.3)	(12.8–13.3)	(13.4–14.9)	(11.3–12.9)	(11.6–12.9)	(11.7–12.3)
MPV (fL)	8.0	7.6	7.3	7.7	7.3	6.6	6.9	7.2	7.5	8.0
	(7.5–8.3)	(7.5–7.8)	(6.7–8.5)	(7.4–7.9)	(6.4–8.0)	(6.4–6.9)	(6.7–7.3)	(6.7–7.7)	(7.5–9.0)	(7.8–8.1)
PDW (%)	15.3	15.4	15.2	17.2	16.1	16.5	16.1	14.5	15.7	15.4
	(13.7–16.9)	(15.2–15.4)	(14.8–16.5)	(16.3–17.8)	(15.2–17.6)	(16.2–16.8)	(15.3–16.7)	(13.7–15.0)	(15.1–17.0)	(15.0–16.1)
LYM (%)	45.2	31.9	41.5	45.7	40.2	32.3	35.3	44.0	41.2	24.5
	(31.5–50.0)	(30.7–36.9)	(26.8–48.7)	(36.4–52.8)	(35.3–43.8)	(27.8–41.9)	(31.2–43.9)	(39.7–45.5)	(34.5–51.5)	(19.7–29.4)
MON (%)	9.4	8.3	9.2	4.7	4.8	5.2	4.6	9.6	8.1	5.6
	(6.9–11.4)	(7.3–9.7)	(6.3–12.5)	(4.4–5.3)	(4.6–5.4)	(3.2–7.3)	(3.8–4.8)	(6.2–13.0)	(6.8–13.7)	(4.1–7.0)
GRA (%)	47.1	60.4	47.2	49.6	54.4	62.4	59.9	46.3	45.7	71.3
	(40.5–59.5)	(53.6–61.7)	(39.4–66.2)	(42.6–58.0)	(51.0–59.8)	(50.8–69.0)	(51.9–64.1)	(42.7–52.8)	(35.5–57.8)	(65.1–74.6)
LYM (10^9^/L)	2.6	2.3	2.6	3.3	3.6	1.9	1.9	2.1	2.3	1.6
	(2.1–2.9)	(1.6–2.9)	(2.1–2.9)	(2.3–4.6)	(2.9–4.3)	(1.6–2.2)	(1.5–2.3)	(1.6–2.8)	(1.8–2.8)	(1.5–1.8)
MON (10^9^/L)	0.5	0.5	0.5	0.3	0.4	0.2	0.1	0.3	0.4	0.3
	(0.5–0.8)	(0.3–0.7)	(0.4–0.7)	(0.2–0.3)	(0.3–0.4)	(0.1–03)	(0.1–0.2)	(0.2–0.6)	(0.3–0.7)	(0.2–0.4)
GRA (10^9^/L)	2.3	4.1	3.2	3.7	5.1	4.0	3.0	2.9	2.7	5.0
	(2.1–4.2)	(3.1–4.8)	(2.5–6.0)	(3.5–3.8)	(4.4–5.9)	(3.0–4.3)	(2.6–3.6)	(2.1–3.3)	(1.8–3.8)	(3.9–6.7)

Data presented as median (Q1–Q3) values. WBC—white blood cell count; RBC—red blood cell count; Hb—hemoglobin; HCT—hematocrit; PLT—platelet count; PCT—plateletcrit; MCV—mean corpuscular volume; MCH—mean corpuscular hemoglobin; MCHC—mean corpuscular hemoglobin concentration; RDW—red blood cell distribution width; MPV—mean platelet volume; PDW—platelet distribution width; LYM—lymphocytes; MON—monocytes; GRA—granulocytes.. The significance of differences between sprint and long-distance groups (FS vs. FL) was calculated using the Mann-Whitney U test. The statistical power of the test was equal to: 0.90 for RBC in 10th week; 0.84 for HGB in 10th week; 0.63 for MCH in 4th week; 0.86, 0.15 and 0.99 for MCHC in 2nd, 3rd and 10th week, respectively; 0.75 for RDW in 10th week; 0.67 for MPV in 4th week; 0.99, 0.06 and 0.92 for LYM (%) in 2nd, 3rd and 10th week respectively; 0.88 and 0.85 for GRA (%) in 2nd and 10th week, respectively. * *p*  <  0.05.

**Table 4:** Male participants’ recovery capillary blood recovery morphology in sprint (MS) and long-distance (ML) groups during 10 weeks of training.

**Variable**	**1st Week**	**2nd Week**	**3rd Week**	**4th Week**	**5th Week**	**6th Week**	**7th Week**	**8th Week**	**9th Week**	**10th Week**
**MS Group**										
WBC (10^9^/L)	6.1	5.3	9.3 *	12.5	5.4	4.7	5.9	6.3	6.2	7.5
	(5.1–6.4)	(4.4–6.6)	(7.6–10.4)	(10.2–13.9)	(4.4–7.5)	(4.2–5.2)	(5.0–6.1)	(5.1–7.9)	(4.6–7.2)	(5.8–8.1)
RBC (10^12^/L)	5.1	4.9	4.4	4.5	4.5 **	4.8 **	4.6	4.4 *	4.9	5.0
	(5.0–5.3)	(4.7–5.3)	(4.1–4.4)	(4.4–4.5)	(4.3–4.7)	(4.7–4.9)	(4.6–5.0)	(4.2–4.8)	(4.8–5.0)	(4.8–5.1)
Hb (mmol/L)	9.0	8.5	8.5	8.5	8.1	8.6	8.3	8.3	9.5 *	9.5
	(8.8–9.2)	(8.3–9.3)	(8.3–8.6)	(8.5–8.8)	(8.0–8.1)	(8.5–8.7)	(8.2–8.6)	(8.0–8.8)	(9.5–9.8)	(9.5–9.7)
HCT (%)	47.9	45.1	39.6	40.1	43.7 *	45.3 *	43.9	40.7 **	45.1 *	45.3
	(46.1–49.4)	(43.7–47.3)	(39.4–40.4)	(39.5–41.7)	(38.2–44.2)	(44.3–45.5)	(43.3–45.2)	(40.1–43.2)	(44.9–46.4)	(44.5–46.2)
PLT (10^9^/L)	224	226	245	276	218	274 *	229	280	213	281 *
	(208–244)	(202–270)	(224–284)	(250–301)	(205–285)	(242–282)	(223–248)	(225–286)	(187–237)	(263–287)
PCT (10^−2^ L/L)	0.17	0.17	0.18	0.21	0.16	0.20 **	0.18	0.20	0.16	0.20
	(0.14–0.20)	(0.15–0.20)	(0.17–0.21)	(0.18–0.22)	(0.15–0.23)	(0.19–0.21)	(0.17–019)	(0.17–0.22)	(0.15–0.17)	(0.17–0.22)
MCV (fL)	91	92	93	93	93	93	94	92	91	92
	(90–95)	(92–93)	(89–93)	(90–95)	(88–94)	(93–93)	(90–95)	(90–94)	(91–92)	(90–93)
MCH (fmol)	1.72	1.78	1.96	1.96	1.76 *	1.79	1.76	1.79	1.95	1.95
	(1.66–1.74)	(1.77–1.80)	(1.93–2.03)	(1.92–2.02)	(1.72–1.93)	(1.78–1.82)	(1.72–1.78)	(1.75–1.99)	(1.92–1.97)	(1.91–1.98)
MCHC (mmol/L)	18.4	19.3	21.3	21.2	18.3	19.1	19.0	19.2 *	21.2	21.1
	(18.3–18.8)	(19.2–19.5)	(21.1–21.5)	(21.1–21.5)	(18.2–21.6)	(19.1–19.3)	(18.8–19.1)	(18.9–21.7)	(21.1–21.4)	(21.0–21.2)
RDW (%)	12.3	12.2	12.6	12.9	12.2	13.0	12.3	12.4	12.3	12.5
	(12.0–12.4)	(12.1–12.3)	(12.1–13.4)	(12.2–12.9)	(11.8–12.6)	(12.8–13.6)	(12.0–12.6)	(12.1–12.5)	(12.2–12.5)	(12.2–12.9)
MPV (fL)	7.5	7.5	7.5	7.3	6.4	7.4	7.5	7.5	7.3	7.0
	(6.9–7.8)	(7.1–7.6)	(7.2–7.6)	(7.1–7.9)	(6.6–7.6)	(7.3–7.5)	(7.0–8.0)	(7.1–7.7)	(6.7–7.6)	(6.6–7.5)
PDW (%)	15.1	14.8	16.9	16.9	14.3	15.5	14.8	16.5	16.8	17.2
	(14.4–16.0)	(14.3–15.1)	(16.4–17.9)	(16.5–17.2)	(14.1–16.5)	(14.9–16.5)	(14.5–15.2)	(15.4–18.2)	(16.2–17.3)	(16.5–17.5)
LYM (%)	38.8	44.0	31.7	39.2	45.3	44.4	40.5	47.5 *	23.7	31.5
	(36.9–43.0)	(42.6–48.9)	(26.5–33.3)	(34.6–42.0)	(40.6–45.8)	(41.8–48.0)	(36.7–40.9)	(44.3–54.6)	(19.8–30.3)	(25.1–37.5)
MON (%)	7.0 *	9.0	4.4	4.6	7.1	7.1	7.5	5.4	13.6	14.8
	(6.3–7.7)	(7.0–10.1)	(3.5–4.8)	(4.3–5.1)	(4.7–8.5)	(6.4–7.6)	(6.4–8.4)	(4.9–6.7)	(12.0–15.7)	(13.1–16.5)
GRA (%)	52.8	47.0	64.1	57.3	49.7 *	50.0	51.7	47.8	65.1	55.4
	(50.0–55.2)	(41.2–49.6)	(62.7–69.7)	(52.6–63.4)	(46.6–51.1)	(45.2–50.4)	(50.4–55.5)	(40.1–48.5)	(52.7–66.0)	(46.0–58.4)
LYM (10^9^/L)	2.3	2.2	2.6	4.7	2.4	1.8	2.1	3.4 *	1.5 *	2.5
	(1.8–2.5)	(1.8–2.9)	(2.3–2.9)	(3.9–5.0)	(2.1–3.4)	(1.5–2.4)	(2.0–2.3)	(2.4–4.2)	(1.3–1.7)	(1.9–3.4)
MON (10^9^/L)	0.4	0.5	0.3	0.5	0.3	0.3	0.4	0.3	0.8	1.0
	(0.3–0.4)	(0.3–0.5)	(0.2–0.4)	(0.4–0.7)	(0.3–0.3)	(0.2–0.3)	(0.3–0.4)	(0.3–0.4)	(0.7–0.8)	(0.9–1.2)
GRA (10^9^/L)	3.2	2.5	5.4	6.3	3.0 *	2.3	3.1	2.8	3.3	3.9
	(2.9–3.5)	(2.1–2.9)	(4.7–6.7)	(5.9–8.5)	(2.3–3.8)	(1.9–2.8)	(2.6–3.5)	(2.6–3.1)	(2.8–5.3)	(2.9–4.6)
**Variable**	**1st Week**	**2nd Week**	**3rd Week**	**4th Week**	**5th Week**	**6th Week**	**7th Week**	**8th Week**	**9th Week**	**10th Week**
**ML Group**										
WBC (10^9^/L)	5.2	5.4	6.2	11.3	7.6	5.2	5.8	5.0	6.7	5.7
	(4.9–5.9)	(5.3–6.6)	(5.8–7.3)	(7.2–12.4)	(6.6–9.0)	(4.5–5.9)	(5.7 -6.6)	(4.8–5.3)	(5.6–7.4)	(5.5–6.4)
RBC (10^12^/L)	4.9	5.2	4.3	4.4	4.2	5.2	4.8	5.0	4.7	5.0
	(4.7–5.0)	(4.9–5.3)	(4.2–4.4)	(4.3–4.6)	(4.0–4.2)	(5.1–5.3)	(4.6–5.4)	(4.8–5.2)	(4.5–5.0)	(4.9–5.2)
Hb (mmol/L)	8.7	9.2	8.4	8.4	8.1	8.8	8.6	8.6	9.3	9.9
	(8.5–8.8)	(8.6–9.4)	(8.1–8.8)	(8.3–9.0)	(8.0–8.6)	(8.5–9.2)	(8.5–8.8)	(8.6–8.9)	(8.6–9.4)	(9.3–10.1)
HCT (%)	45.3	47.4	39.4	40.2	38.2	46.7	44.9	47.7	43.9	46.6
	(42.3–46.1)	(44.3–48.5)	(38.9–40.3)	(39.6–42.3)	(36.9–39.6)	(46.2–48.4)	(44.3–47.0)	(46.3–47.9)	(40.0–44.4)	(44.9–47.5)
PLT (10^9^/L)	251	252	238	321	260	218	214	231	229	199
	(195–257)	(214–287)	(228–274)	(285–373)	(233–303)	(169–231)	(209–254)	(221–291)	(214–240)	(177–255)
PCT (10^−2^ L/L)	0.18	0.18	0.19	0.23	0.19	0.15	0.18	0.18	0.17	0.16
	(0.14–0.21)	(0.15–0.20)	(0.16–0.21)	(0.21–0.29)	(0.17–0.21)	(0.13–0.17)	(0.16–0.18)	(0.17–0.21)	(0.15–017)	(0.13–0.18)
MCV (fL)	93	91	93	93	93	91	92	93	92	91
	(90–94)	(89–93)	(90–95)	(91–96)	(92–95)	(88–92)	(91–94)	(91–95)	(88–93)	(91–93)
MCH (fmol)	1.77	1.78	1.93	1.97	2.02	1.71	1.73	1.72	1.95	1.94
	(1.73–1.80)	(1.70–1.80)	(1.90–2.01)	(1.88–2.04)	(1.95–2.05)	(1.64–1.78)	(1.67–1.84)	(1.69–1.77)	(1.81–1.97)	(1.89–1.97)
MCHC (mmol/L)	18.8	19.4	21.1	20.9	21.5	18.6	18.8	18.6	21.3	21.2
	(18.6–20.2)	(19.2–19.6)	(20.9–21.3)	(20.8–21.2)	(21.3–21.7)	(18.3–18.8)	(18.7–19.6)	(18.5–18.7)	(21.0–21.3)	(21.0–21.4)
RDW (%)	12.6	12.3	12.3	12.7	12.5	12.8	12.1	12.4	12.6	12.5
	(12.1–13.2)	(11.8–12.3)	(12.0–12.5)	(12.2–12.8)	(12.1–12.6)	(12.3–13.3)	(12.0–12.7)	(12.2–13.3)	(12.3–13.2)	(12.2–12.6)
MPV (fL)	7.5	7.1	7.7	7.2	7.4	7.3	7.7	7.5	7.2	7.6
	(7.2–8.0)	(6.8–7.3)	(6.6–8.1)	(6.7–7.9)	(6.8–7.5)	(7.0–8.0)	(7.3–8.4)	(7.4–7.6)	(7.1–7.6)	(7.2–8.1)
PDW (%)	15.0	12.7	16.7	16.9	16.4	15.7	15.0	15.5	17.0	16.4
	(14.7–15.8)	(13.7–15.1)	(16.2–17.7)	(16.1–17.0)	(15.7–16.9)	(13.7–15.9)	(14.6–15.5)	(15.2–16.0)	(15.6–17.2)	(15.8–16.8)
LYM (%)	43.0	46.6	39.1	38.5	39.0	42.7	35.8	40.9	32.5	30.3
	(36.8–45.3)	(40.1–52.3)	(33.2–41.0)	(32.3–50.7)	(34.6–44.1)	(38.5–49.0)	(32.8–39.0)	(34.4–45.6)	(23.6–41.1)	(24.9–31.7)
MON (%)	10.5	9.9	4.2	5.1	4.8	8.2	6.8	6.7	11.1	16.7
	(8.4–10.9)	(8.6–11.0)	(4.1–4.3)	(4.4–5.2)	(3.9–5.1)	(5.3–8.9)	(5.5–7.3)	(5.5–7.1)	(5.7–13.6)	(15.0–19.0)
GRA (%)	46.2	43.7	56.8	56.4	56.1	49.1	58.6	50.3	56.1	51.4
	(43.8–54.8)	(39.1–49.3)	(51.7–62.3)	(44.2–63.3)	(51.4–61.7)	(42.1–55.2)	(52.8–60.4)	(48.9–60.5)	(51.9–63.6)	(48.0–61.5)
LYM (10^9^/L)	2.0	2.7	2.4	4.7	2.8	1.9	1.9	2.0	2.0	1.5
	(1.8–2.3)	(2.4–3.2)	(1.90–2.80)	(3.5–53)	(2.4–3.0)	(1.7–2.6)	(1.7–2.5)	(1.5–2.4)	(1.7–2.3)	(1.4–1.7)
MON (10^9^/L)	0.5	0.5	0.2	0.6	0.3	0.3	0.4	0.3	0.7	1.0
	(0.4–0.6)	(0.4–0.7)	(0.2–0.3)	(0.3–0.6)	(0.2–0.3)	(0.2–0.4)	(0.3–04)	(0.2–0.3)	(0.4–0.7)	(0.8–1.0)
GRA (10^9^/L)	2.5	2.4	3.7	5.2	5.0	2.6	3.6	2.8	4.0	3.0
	(2.4–3.3)	(1.9–3.5)	(3.2–5.4)	(4.6–7.1)	(3.5–5.7)	(2.3–2.9)	(3.4–3.8)	(2.5–2.9)	(2.9–4.5)	(2.7–3.9)

Data presented as median (Q1–Q3) values. WBC—white blood cell count; RBC—red blood cell count; Hb—hemoglobin; HCT—hematocrit; PLT—platelet count; PCT—plateletcrit; MCV—mean corpuscular volume; MCH—mean corpuscular hemoglobin; MCHC—mean corpuscular hemoglobin concentration; RDW—red blood cell distribution width; MPV—mean platelet volume; PDW—platelet distribution width; LYM—lymphocytes; MON—monocytes; GRA—granulocytes. The significance of differences between sprint and long distance groups (MS vs. ML) was calculated using the Mann-Whitney U test. The statistical power of the test was equal to: 0.69 for WBC in 3rd week; 0.89, 0.76 and 0.76 for RBC in 5th, 6th and 8th week, respectively; 0.62 for HGB in 9th week; 0.71, 0.73, 0.91 and 0.18 for HCT in 5th, 6th, 8th and 9th week, respectively; 0.65 and 0.66 for PLT in 6th and 10th week, respectively; 0.97 for PCT in 6th week; 0.73 for MCH in 5th week; 0.76 for MCHC in 8th week; 0.59 for LYM (%) in 8th week; 0.64 in MON (%) in 1st week; 0.81 in GRA (%) in 5th week; 0.75 and 0.67 for LYM (10^9^/L) in 8th and 9th week, respectively; 0.74 for GRA (10^9^/L) in 5th week. * *p*  <  0.05; ** *p*  <  0.01.

## Data Availability

The datasets generated and/or analyzed during the current study are available from the corresponding author on reasonable request.

## Supplementary Materials

The following supporting information can be downloaded at: https://www.mdpi.com/article/10.3390/ijerph19148580/s1, Table S1: Participants’ training details; Table S2: GLMM analysis results.
