# Supplementary material for: Capillary Blood Recovery Variables in Young Swimmers: An Observational Case Study
Source: Int J Environ Res Public Health. 2022 Jul 14;19(14):8580. doi: 10.3390/ijerph19148580 (PMC9318784; doi:10.3390/ijerph19148580)
Supplement: Supplementary file 1 [file ijerph-19-08580-s001.zip › ijerph-1768756-supplementary.pdf]

Table S1. Participants' training details.

Variable	1st week	2nd week	3rd week	4th week	5th week	6th week	7th week	8th week	9th week	10th week
**FS group**										
Distance swam (km)	18.0 (14.5 – 19.0)	29.0 (19.0 – 31.0)	32.0 (20.0 – 36.5)	35.0 (32.0 – 39.1)	33.5 (25.0 – 39.0)	43.0 (40.0 – 51.0)	43.0 (39.0 – 53.0)	38.0 (26.0 – 38.5)	42.0 (33.0 – 43.5)	38.5 (28.5 – 50.0)
Water training sessions	6 (6 – 6)	8 (7 – 9)	9 (4 – 9)	9 (7 – 10)	8 (6 – 9)	10 (10 – 10)	10 (10 – 10)	10 (7 – 10)	10 (10 – 11)	9 (9 – 10)
Dry-land trainings (h)	8 (5 – 9)	6 (2 – 7)	5 (4 – 6)	5 (3 – 6)	5 (2 – 5)	5 (4 – 5)	5 (3 – 5)	5 (2 – 6)	4 (4 – 5)	4 (3 – 6)
**FL group**										
Distance swam (km)	33.6 (28.1 – 36.0)	33.6 (28.1 – 36.0)	42.0 (33.8 – 43.6)	33.6 (28.1 – 36.0)	28.1 (20.0 – 40.3)	42.0 (33.8 – 43.6)	28.1 (20.0 – 40.3)	28.1 (20.0 – 40.3)	46.45 (24.2 – 51.4)	46.4 (24.2 – 51.4)
Water training sessions	6 (6 – 6)	10 (10 – 10)	9 (9 – 9)	10 (10 – 10)	8 (5 – 8)	10 (10 – 10)	9 (5 – 10)	10 (9 – 10)	12 (11 – 12)	11 (9 – 11)
Dry-land trainings (h)	7 (7 – 7)	8 (5 – 8)	7 (5 – 9)	9 (9 – 10)	4 (4 – 6)	5 (4 – 5)	8 (4 – 8)	7 (6 – 8)	8 (8 – 8)	6 (6 – 6)
**MS group**										
Distance swam (km)	28.0 (19.0 – 38.5)	35.0 (20.0 – 38.9)	42.7 (20.0 – 48.0)	35.0 (23.4 – 35.0)	39.0 (36.3 – 43.0)	32.5 (30.7 – 51.0)	24.5 (23.4 – 37.0)	36.3 (25.7 – 37.5)	30.7 (25.7 – 42.4)	29.3 (28.5 – 29.3)
Water training sessions	7 (6 – 9)	8 (5 – 9)	8 (4 – 9)	5 (5 – 7)	8 (8 – 11)	10 (7 – 10)	6 (5 – 9)	9 (8 – 10)	10 (7 – 10)	8 (7 – 8)
Dry-land trainings (h)	8 (4 – 10)	8 (4 – 10)	8 (4 – 9)	8 (8 – 9)	9 (8 – 10)	8 (7 – 12)	8 (5 – 9)	7 (6 – 10)	6 (6 – 10)	8 (5 – 12)
**ML group**										
Distance swam (km)	23.3 (22.0 – 46.1)	54.4 (48.4 – 54.4)	54.4 (30.3 – 54.4)	53.6 (30.9 – 54.4)	48.6 (29.5 – 48.6)	60.2 (24.0 – 61.2)	53.1 (29.5 – 59.2)	52.2 (26.9 – 52.2)	57.4 (44.4 – 57.5)	53.8 (26.1 – 58.8)
Water training sessions	6 (6 – 11)	10 (10 – 10)	9 (7 – 11)	10 (7 – 10)	8 (5 – 9)	10 (5 – 10)	10 (5 – 11)	9 (5 – 10)	11 (9 – 12)	9 (7 – 11)
Dry-land trainings (h)	10 (7 – 12)	5 (3 – 6)	7 (4 – 8)	8 (7 – 10)	5 (3 – 6)	6 (4 – 8)	5 (2 – 8)	4 (4 – 9)	5 (4 – 8)	7 (4 – 8)

Data presented as median (min–max) values.

Table S2. GLMM analysis results.

Variable	Females	Males
	FDR-adjusted *p*	
CK	0.216	0.380
AST	0.160	0.313
ALT	0.166	0.398
ALP	0.700	0.067
LDH	0.160	<0.0001
urea	0.227	0.186
creatinine	0.060	0.274
Total bilirubin	0.180	0.067
Direct bilirubin	0.992	0.001
CRP	0.259	0.001
ferritin	0.298	0.068
iron	0.569	0.093
WBC	0.166	0.006
RBC	0.180	0.001
Hb	0.060	0.121
HCT	0.280	<0.0001
PLT	0.569	0.048
PCT	0.660	0.065
MCV	0.237	0.541
MCH	0.180	0.068
MCHC	0.001	<0.0001
RDW	0.075	0.165
MPV	0.166	0.436
PDW	0.075	0.201
LYM (%)	0.093	0.067
MON (%)	0.667	0.001
GRA (%)	0.075	0.279
LYM absolute count	0.259	0.014
MON absolute count	0.180	0.068
GRA absolute count	0.114	0.017

CK - creatine kinase; AST - aspartate aminotransferase; ALT - alanine aminotransferase; ALP - alkaline phosphatase; LDH - lactate dehydrogenase; CRP - C-reactive protein; WBC - white blood cell count; RBC - red blood cell count; Hb - hemoglobin; HCT - hematocrit; PLT - platelet count; PCT - plateletcrit; MCV - mean corpuscular volume; MCH - mean corpuscular hemoglobin; MCHC - mean corpuscular hemoglobin concentration; RDW - red blood cell distribution width; MPV - mean platelet volume; PDW - platelet distribution width; LYM - lymphocytes; MON - monocytes; GRA – granulocytes; FDR - False Discovery Rate.
